# Post-marketing safety surveillance and re-evaluaiton of Shu-Xue-Ning injection: a real-world study based on 30,122 cases

**DOI:** 10.3389/fphar.2023.1194367

**Published:** 2023-11-29

**Authors:** Jin Xinyao, Zhang Yifan, Wang Keyi, Pang Wentai, Wang Chunyang, Wang Hui, Liu Chunxiang, Xue Yunhua, Zheng Wenke

**Affiliations:** ^1^ Center for Evidence-Based Medicine, Tianjin University of Traditional Chinese Medicine, Tianjin, China; ^2^ School of Statistics and Data Science, Nankai University, Tianjin, China; ^3^ School of Mathematical Sciences, Nankai University, Tianjin, China

**Keywords:** Shineway^®^ Shu-Xue-Ning injection, TCM injection, adverse reactions, intensive hospital monitoring, real-world study

## Abstract

**Objective:** This study aims to investigate the safety of Shu-Xue-Ning injection (SXNI) in real-world clinical applications.

**Methods:** A prospective, multi-center, large-sample intensive monitoring method was used to monitor the use of SXNI in several medical institutions across China while collecting patients’ dosing and adverse event information. Patients who suspected as adverse reactions made comparisons with patients who did not report adverse reactions to calculate the correlation between relevant risk factors and suspected adverse reactions. Statistical analysis software SAS 9.1 was used for data analysis.

**Results:** A total of 48 hospitals participated in this intensive monitoring study of SXNI, and 30,122 patients were monitored from July 2015 to December 2018. A total of 1,908 adverse events were reported during the use of SXNI, with an adverse event rate of 6.33% and a 95% confidence interval (CI) of 6.06%–6.61%. Association assessment showed that 54 cases presented with SXNI-related adverse reactions with an incidence of 0.18% and a 95% CI of 0.13%–0.23%, thereby indicating that the incidence of SXNI-related adverse reactions was occasional. SXNI-related adverse reactions involved 9 systems-organs with 20 clinical manifestations, and the most common adverse reactions were rash, pruritus, and other damages of skin and its appendages. No serious adverse reactions were observed; 27.78% of the adverse reactions occurred within 30 min of drug administration and more than half of them occurred within 2 h of drug administration; 96.3% of the adverse reactions were cured or improved. Causal analysis showed that women, long dispensing time, and slow dripping speed rate were considered as risk factors.

**Conclusion:** The incidence of SXNI-related adverse reactions in real-world clinical applications is occasional and in a reasonable range with a good prognosis.

## 1 Introduction

Traditional Chinese medicine (TCM) injections are a landmark achievement in the modernization of TCM and have advantages of high bio availability and fast onset of action (Wang and Lai, 2005). TCM injections have gained wide applications, but its safety issue is of great concern (Gong et al., 2017). The *National Annual Monitoring Report of Adverse Drug Reactions* (2020) shows that the National Monitoring Network of Adverse Drug Reactions received 1,676,000 *Adverse Drug Reaction/Event Report Forms* in 2020, of which chemical drugs accounted for 83.0% and TCM accounted for 13.4%; injections accounted for 60.4% and 33.3% of the chemical drug-related and TCM-related adverse reaction/event reports, respectively (State Drug Administration Drug Assessment Center, 2020). It is evident that the safety of TCM injections is not necessarily lower than that of chemical injections, and the safety events in TCM injections may be attributed to drug properties, irrational drug use, and individual patient differences (Liang and Li, 2007; Zhang, 2014).

Systematic post-marketing pharmaco-epidemiological studies are lacking for some TCM injections, which results in a relative shortage of data on the incidence of adverse reactions, and a relatively poor understanding of clinical manifestations of adverse reactions. In order to further improve the safety of TCM injections, the State Food and Drug Administration of China issued the *Notice on the Re-assessment of the Safety of TCM injections* in 2009 to initiate re-assessment of the safety of TCM injections (Zhang, 2009).

The national 12th Five-Year Plan of drug safety also proposes to carry out safety risk analysis and assessment of TCM injections (State Council, 2012). Later, the State Council issued the *Opinions on Deepening the Review and Approval System Reform to Encourage Innovation in Drugs and Medical Devices* in 2017 proposing to re-evaluate the marketed drug injections as well. Currently, the clinical safety of several TCM injection products, such as Xuesaitong injections (lyophilized) and Xueshuantong injections (lyophilized), have been intensively monitored for more than 30,000 cases (Wang et al., 2015; Li et al., 2018; Jin et al., 2020). A systematic assessment based on an intensive, clinical safety monitoring study of TCM injections revealed that, as of February 2018, a total of 296,200 patients had been monitored for 14 TCM injection products with an overall incidence of adverse reactions of 1.57 per 1,000 (Li et al., 2019).

The Shu-Xue-Ning injection (SXNI) is a sterilized aqueous solution of Ginkgo biloba leaf extract made by Shineway Pharmaceutical Group Co., Ltd. It’s a traditional Chinese patent medicine, according to the package inserts of drug, its specification is 5 mL per tube, equivalent to 17.5 mg of ginkgo biloba extract (containing 4.2 mg of total flavonoids; containing 0.70 mg of ginkgolides). It has been used in clinical for many years and can ensure uniform and stable composition between batches. SXNI used in the clinical treatment of ischemic cardiovascular, cerebrovascular diseases, angina pectoris, coronary heart disease, cerebral embolism, and cerebral vasospasm (Ge et al., 2012; Tao and Chen, 2012; Du et al., 2017; Wan et al., 2018). The main active constituents of SXNI are total flavonoids (Quercetin, Kaempferin, Isorhamnetin) and terpene lactones (Ginkgolide A, Ginkgolide B, Ginkgolide C, and Bilobalide) (Ou et al., 2010).

Since the start of the re-assessment work, quality control standards of SXNI has been established in all aspects from the production of raw materials to product processing, which has improved the stability and controllability of the product (Zhao et al., 2015). A post-marketing re-assessment of SXNI was conducted herein using an intensive hospital monitoring approach to systematically assess the safety of this injection for a wide range of populations in a real-world clinical setting.

SXNI is commonly used in clinical practice and has previously been completed reevaluation throng post-marketing safety based on real-world and evidence-based evaluations (Wang et al., 2018). Compared with previous study, this study were only includes pantients who used SXNI produced by Shineway and the data were completely based on real-world observations, with a much larger sample size than previous studies.

## 2 Data and methods

### 2.1 Study design

**FIGURE 1:**
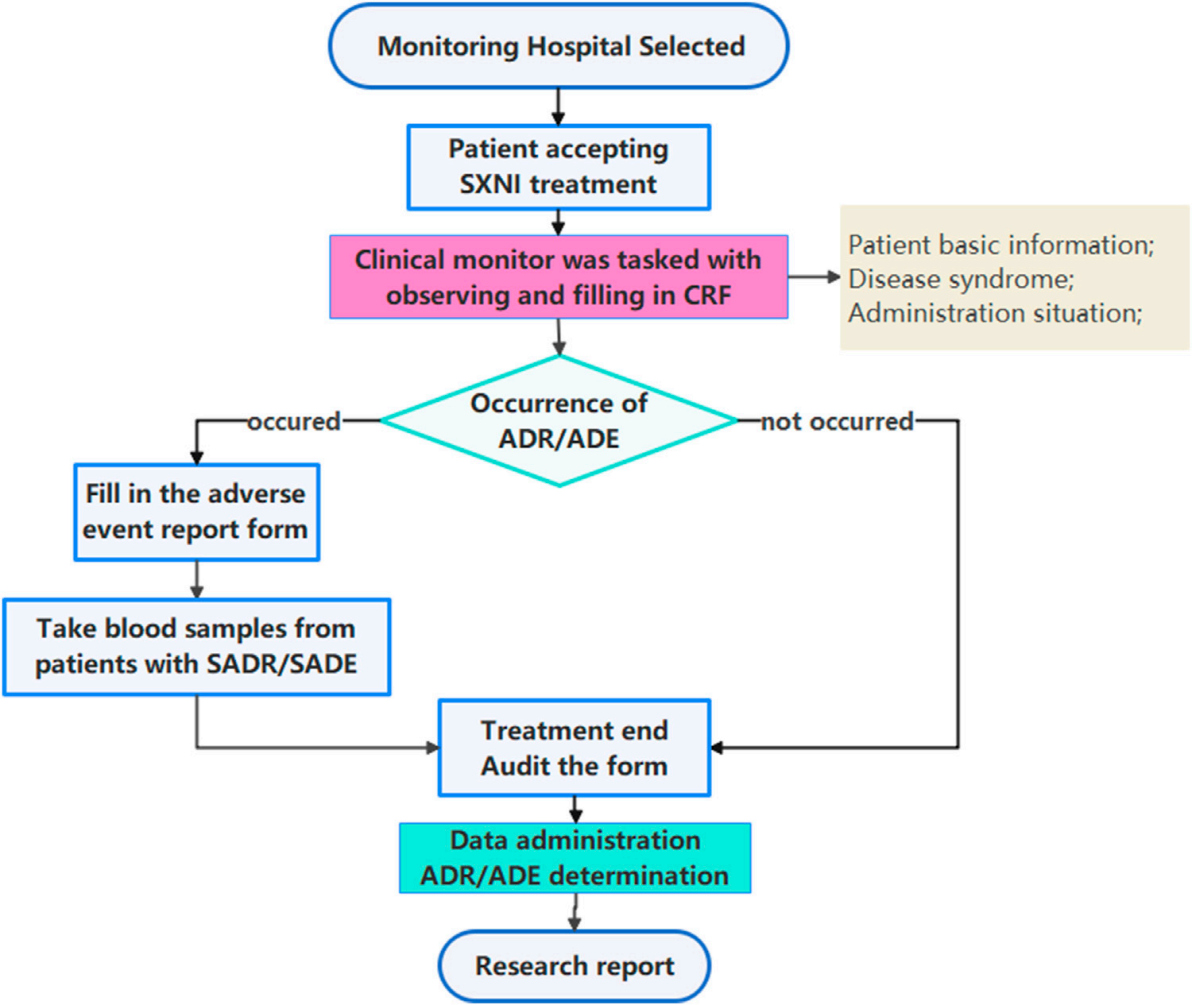
Post-marketing safety surveillance and re-evaluation of SXNI workflow.

This study adopted a prospective, multicenter, large-sample intensive monitoring design. The clinical trial registration number was ChiCTR-OPC-15006649, which was approved by the Medical Ethics Committee of Tianjin University of Traditional Chinese Medicine (TJUTCM-EC20150005). This was a retrospective study without intervening in the diagnosis and treatment of patients, adhered to the Declaration of Helsinki and was approved by ethics committee to waive the patients' informed consent. This observational study was reported based on the STROBE (STrengthening the Reporting of OBservational studies in Epidemiology) statement (von Elm et al., 2014). Post-marketing safety re-evaluation of SXNI workflow was shown in Figure 1.

### 2.2 Monitoring institutions

Considering factors such as hospital type, hospital grade, and geographical location, 48 hospitals (secondary Grade-A or above) were selected nationwide as intensive monitoring institutions.

### 2.3 Inclusion and exclusion criteria

Inclusion criteria: In-patients who received at least 1 SXNI treatment in the monitoring institution (regardless of the departments) were included in this study, regardless of patient age, gender, disease type, or condition.

Exclusion criteria: patients who did not use SXNI.

### 2.4 Sample size calculation

The reported incidence of adverse reactions to TCM injections is mostly occasional (0.1%, 1%) and rare (0.01%, 0.1%) (Li et al., 2018). The number of subjects should be 30,000 when assuming a 95% probability of finding at least one rare adverse reaction (Zhang et al., 2017).

### 2.5 Monitoring cycle

The monitoring cycle ranged from July 2015 to December 2018.

### 2.6 Monitoring content

In view of the prior research experiences of intensive hospital monitoring and the characteristics of SXNI, this study determined that the information to be collected was basic patient information, SXNI production and administration-related information, and SXNI safety-related information. Basic patient information comprised gender and age; SXNI production and administration-related information comprised production batch number, drug specification, usage, dosage, treatment course, combined medication, and change of treatment regimen. SXNI safety-related information comprised the incidence of adverse reactions/events, clinical manifestations of adverse reactions, types of adverse reactions, severity of adverse reactions, treatment measures of adverse reactions, and prognosis.

### 2.7 Data acquisition and management

In each participating hospital, data were collected using Case Reporting Form (CRF) and Adverse Event Reporting Form (AERF). Basic patient information and medication information were retrieved directly from the medical record of patients without interfering with the medication treatment conducted by the responsible clinical physician and without affecting the normal treatment of the patient, where a clinical monitor was tasked with observing and filling in CRF, as well as filling in AERF if an adverse event occurred. A clinical inspector was responsible for data verification and data transfer to ensure quality control of the intensive monitoring process.

After confirming that the CRF was completed in a timely, accurate, and complete manner, the clinical inspector signed with the clinical monitor and transmitted the CRF data to the online electronic data management system. The medical data manager revised the medical event names to standard terms with reference to WHO-ART and verified them with the monitor while standardizing the information of doctors’ orders, such as the names of chemical and proprietary Chinese medicines. The database was locked upon verification of the information, followed by statistical analysis.

### 2.8 Quality control

The Institute of Clinical Assessment of Tianjin University of Traditional Chinese Medicine organized program training for the project managers of all participating hospitals before the start of monitoring in the participating hospitals. This included interpretation of study protocol, filling out CRF and AERF, and judgment and association assessment of adverse reactions/events. Beijing Excellence Future International Consulting Co., Ltd. assigned inspectors to train all monitors in the participating hospitals on the study protocol and the completion of CRF and AERF. Primary quality control was the responsibility of the participating hospitals, who were tasked with inspecting the quality of monitored cases, including the study progress, all original data verification, authenticity verification, and electronic data reporting. The project leader of the hospital should review and sign the quality checklist every week and take appropriate measures to overcome the existing quality problems. For secondary quality control, inspectors from Beijing Excellence Future International Consulting Co., Ltd. regularly monitored each monitoring hospital to ensure that the CRF data were true, accurate, and complete. They verified the same with the electronic data management system data, completed the monitoring report, and signed it. For tertiary quality control, auditors from the Institute of Clinical Assessment of Tianjin University of Traditional Chinese Medicine regularly audited the monitored cases in the participating hospitals, completed the audit report, and signed it.

### 2.9 Association assessment of adverse events

After the data were verified and the database was locked, the research team set up an expert committee of adverse event assessment with three to five experts selected from the assessment expert pool. The association was initially determined by the monitor and finally determined by the expert committee.

### 2.10 Statistical analysis

Data analysis was performed using software SAS 9.1. Measurement data such as age were expressed as mean, standard deviation, minimum, and maximum values. Categorical variables such as gender, drug allergy history, and the occurrence of adverse reactions were expressed as their frequencies and composition ratios. For hypothesis tests, differences were considered statistically significant at *p* < 0.05. The type and incidence of SXNI-related adverse reactions/events were expressed as percentages and 95% confidence intervals (CI), respectively. Pearson’s chi-square test, Permutation test and Logistic regression was used to compare the classified data on correlation analysis.

## 3 Results

### 3.1 Monitoring units and patient inclusion

As detailed in Table 1, a total of 48 medical institutions participated in this study, monitoring 30,122 patients, with a sample size of 11 to 2,021 cases monitored at each institution.

**TABLE 1:** Distribution of monitoring institutions and the patients.

Serial number	Province/municipality	Institution name	Number of cases	Composition ratio (%)
1	Shanxi	Shanxi Cardiovascular Disease Hospital	2021	6.71
2	Henan	Luoyang First People’s Hospital	1997	6.63
3	Liaoning	Panjin Central Hospital	1800	5.98
4	Hebei	Handan Central Hospital	1500	4.98
5	Henan	The First Affiliated Hospital of Xinxiang Medical College	1500	4.98
6	Jilin	Jilin City People’s Hospital	1300	4.32
7	Liaoning	Shenyang Medical College Shenzhou Hospital	1290	4.28
8	Zhejiang	Zhejiang Xinhua Hospital	1144	3.8
9	Shaanxi	The Second People’s Hospital of Shaanxi Province	1000	3.32
10	Heilongjiang	The First Hospital of Heilongjiang University of Traditional Chinese Medicine	996	3.31
11	Jilin	Jilin University Second Hospital	950	3.15
12	Sichuan	Zhongjiang County People’s Hospital	900	2.99
13	Jilin	General Hospital of Jihua Group Corporation	883	2.93
14	Shaanxi	The Fourth People’s Hospital of Shaanxi Province	800	2.66
15	Shanxi	Shanxi Provincial People’s Hospital	793	2.63
16	Hebei	Shijiazhuang City Hospital of Traditional Chinese Medicine	761	2.53
17	Zhejiang	Zhejiang Hospital of Traditional Chinese Medicine	700	2.32
18	Shaanxi	Xi’an High-Tech Hospital	700	2.32
19	Hebei	Hebei Provincial Hospital of Traditional Chinese Medicine	615	2.04
20	Henan	Henan Provincial Hospital of Traditional Chinese Medicine	594	1.97
21	Shanghai	Shanghai Renji Hospital	525	1.74
22	Zhejiang	Yuhang District First People’s Hospital	502	1.67
23	Hebei	Tangshan Hospital of Traditional Chinese Medicine	501	1.66
24	Shanxi	Taiyuan Second People’s Hospital	497	1.65
25	Henan	Hebi First People’s Hospital	452	1.5
26	Heilongjiang	The Second Hospital of Heilongjiang University of Traditional Chinese Medicine	450	1.49
27	Jilin	Jilin Provincial People’s Hospital	435	1.44
28	Beijing	The Central Hospital of China Aerospace Corporation	413	1.37
29	Hebei	Handan City People’s Hospital	400	1.33
30	Hebei	The Second Hospital of Hebei Medical University	388	1.29
31	Henan	Luoyang Oriental Hospital	355	1.18
32	Heilongjiang	Qiqihar First Hospital	350	1.16
33	Hebei	Yi County Hospital of Traditional Chinese Medicine	350	1.16
34	Beijing	Oriental Hospital of Beijing University of Traditional Chinese Medicine	338	1.12
35	Tianjin	The Second Affiliated Hospital of Tianjin University of Traditional Chinese Medicine	275	0.91
36	Chongqing	Chongqing Fifth People’s Hospital	255	0.85
37	Hebei	Baoding Hospital of Integrative Medicine	245	0.81
38	Hebei	Qinghe County Central Hospital	200	0.66
39	Liaoning	Liaoning University of Chinese Medicine Affiliated Hospital	200	0.66
40	Hebei	Hebei Provincial People’s Hospital	182	0.6
41	Liaoning	The Second Hospital of Liaoning University of Traditional Chinese Medicine	128	0.42
42	Hebei	Jingmen First People’s Hospital	105	0.35
43	Sichuan	Chengdu Armed Police Hospital	100	0.33
44	Chongqing	Chongqing Third People’s Hospital	87	0.29
45	Hebei	Shijiazhuang Eighth Hospital	60	0.2
46	Shanxi	Shanxi Linfen Hospital	43	0.14
47	Hunan	The First Hospital of South China University	31	0.1
48	Shanxi	The First Hospital of Shanxi Medical University	11	0.04
Total			30122	100.00

There were 7 secondary hospitals and 41 tertiary hospitals monitoring 2,652 (8.8%) and 27,470 (91.2%) of the total number, respectively. There were 34 Western medicine hospitals monitoring 22,825 patients (75.8%) and 13 TCM hospitals monitoring 7,052 patients (23.4%), with 1 TCM and Western medicine-combined hospital monitoring 245 patients (0.8%). Of the 48 hospitals, 19 are in North China monitoring 9,593 patients (31.85%), 11 in Northeast China monitoring 8,782 patients (29.15%), 7 in Central China monitoring 5,034 patients (16.71%), 4 in East China monitoring 2,871 patients (9.53%), 3 in Northwest China monitoring 2,500 patients (8.30%), and 4 in Southwest China monitoring 1,342 patients (4.46%).

The mean age of the included patients was 62.63 ± 14.85 years, with the youngest and oldest ages being 3 and 103 years, respectively; the mean height and mean body weight were 165.29 ± 8.63 cm and 66.24 ± 11.78 kg, respectively. Of the patients, 15,600 (51.79%) were male and 14,522 (48.21%) were female. There were 5,326 patients (17.68%) with a habit of smoking and 3,676 patients (12.20%) with a habit of drinking. A total of 1,013 cases (3.36%) had a history of adverse reactions. Clinical characteristics of all patients and patient groups according to adverse reactions were showed in Table 2.

**TABLE 2:** Clinical characteristics of all patients and patient groups according to adverse reactions.

Clinical features	Overall (*N* = 30122)	Adverse reactions group (*N* = 54)	Non-adverse reactions group (*N* = 30068)
Age [years, M±S]	59.33 ± 14.52	56.28 ± 14.24	61.41 ± 14.34
Sex [n (%)]			
Female	14522(48.21)	38 (70.37)	14484 (48.17)
Male	15600(51.79)	16(29.63)	15584(51.83)
Storage period [min, M±S (%)]	17.44 ± 11.96	22.85 ± 13.71	17.43 ± 11.96
Treatment course [d, M±S (%)]	9.07 ± 5.56	5.00 ± 4.86	9.07 ± 5.56
Dripping speed [Drips per minute, M±S (%)]	46.71 ± 15.68	42.28 ± 13.42	46.72 ± 15.69

### 3.2 Adverse events and association assessment

A total of 1,908 adverse events were reported in the study, with an adverse event rate of 6.33% and 95% CI of 6.06%–6.61%. The incidence of SXNI-related adverse reactions was 0.18%, with 95% CI ranging from 0.13% to 0.23%.

### 3.3 Clinical manifestations of adverse reactions

There were 20 clinical manifestations of SXNI-related adverse reactions involving 9 systems/organs. Among them, 33 cases presented with skin and appendage damage, for which the most common manifestations were pruritus and rash, while 21 cases presented with central and nervous system damage, for which the common manifestations were dizziness and headache. The results are summarized in Table 3.

**TABLE 3:** Systems/organs involved in SXNI-related adverse reactions and their clinical manifestations.

Involved systems/organs	Frequency (composition ratio %)	Clinical manifestations (frequency)
Skin and its appendage damage	33 (42.31)	Pruritus (16), rash (14), erythematous rash (1), angioneurotic edema (1), purpuric rash (1)
Central and peripheral nervous system damage	21 (26.92)	Dizziness (10), headache (10), local numbness (1)
Systemic damage	10 (12.82)	Fever (4), malaise (2), weakness (2), chills (2)
Heart rate and rhythm disturbances	5 (6.41)	Heart palpitations (5)
Respiratory damage	3 (3.85)	Dyspnea (2), laryngeal edema (1)
Sympathetic parasympathetic nervous system damage	2 (2.56)	Flushing (1), wet and cold skin (1)
Gastrointestinal system damage	2 (2.56)	Nausea (2)
General cardiovascular system damage	1 (1.28)	Hypertension (1)
Medication site damage	1 (1.28)	Injection site rash (1)

### 3.4 Time from drug administration to the onset of adverse reactions

In this study, SXNI-related adverse reactions were found to occur within 7 days of dosing, including 27.78% within 30 min and 83.33% within 24 h. The results are summarized in Table 4.

**TABLE 4:** Time from drug administration to the onset of adverse reactions.

Time	Frequency (number of cases)	Composition ratio (%)
0–30 min	15	27.78
31 min–2 h	16	29.63
2–24 h	14	25.92
1–7 d	9	16.67
Total	54	100

### 3.5 Types of adverse reactions and outcomes

According to the definition of adverse reactions in the *Administrative Measures for the Reporting and Monitoring of Adverse Drug Reactions*, no serious adverse reactions were found in this study. According to the description of clinical manifestations in SXNI instructions (General Office of the State Food and Drug Administration, 2013), three general adverse reactions were observed: discomfort, weakness, and local numbness.

We observed that 39 cases of adverse reactions were cured, which accounted for 72.22% of the total number of adverse reactions; 13 cases of adverse reactions were improved, which accounted for 24.08%, and no adverse reactions led to death. The results are summarized in Table 5.

**TABLE 5:** Outcomes of adverse reactions.

Outcome	Frequency (number of cases)	Composition ratio (%)
Cured	39	72.22
Improved	13	24.08
Unknown	2	3.70
Total	54	100.00

### 3.6 Age and gender of patients with adverse reactions

Among the patients with adverse reactions, 38 were female accounting for 70.37%, and 16 were male accounting for 29.63%. Patients with adverse reactions were predominantly middle-aged and elderly, which was consistent with the age of onset of cardiovascular and cerebrovascular diseases. The results are summarized in Table 6.

**TABLE 6:** Age distribution of patients with adverse reactions.

Age (years)	Frequency (number of cases)	Composition ratio (%)
≤ 18	0	0.00
19–45	2	3.70
46–65	26	48.15
66–80	16	29.63
≥ 81	10	18.52
Total	54	100.00

### 3.7 History of allergy in patients with adverse reactions

Among the 54 patients with adverse reactions, 34 patients (62.96%) had no history of allergy, 4 (7.41%) had an unknown history of food/drug allergy, and 16 (29.63%) had a history of food/drug allergy. There were 12 patients with a history of drug allergy who were allergic to cephalosporin antibiotics, penicillin, and sulfonamides, and 2 patients with food allergy were allergic to seafood; 2 patients had a history of both food and drug allergies.

### 3.8 Dosing characteristics of SXNI

Fifty-four patients with adverse reactions were administered by intravenous drip, of whom 25 were first-time SXNI users, 1 had a history of SXNI treatment, and 28 had an unknown history of SXNI treatment. The single dose was 10 mL in 3 cases, 15 mL in 2 cases, 20 mL in 48 cases, and 25 mL in 1 case, with 88.89% of the patients qualifying for the intravenous drip dose of 20 mL per day as required in the SXNI instructions.

The solvent dose was 100 mL in 6 cases, 150 mL in 1 case, 200 mL in 2 cases, and 250 mL in 45 cases; the solvent type was 0.9% normal saline in 32 cases, 10% glucose in 1 case, 5% glucose in 20 cases, and 5% xylitol in 1 case. A total of 18 cases qualified for the intravenous drip dose of 250 mL or 500 mL in 5% glucose as required in the SXNI instructions, thereby accounting for 33.33%.

The drip rate ranged from as fast as 75 drops/min to as slow as 20 drops/min, with a mean of 42.28 ± 13.42 drops/min. After dispensing, the injection mix was allowed to settle for 0–60 min, with a mean of 22.85 ± 13.71 min.

### 3.9 Combined medications in patients with adverse reactions

The top 10 drugs in combined medications in 54 patients who experienced adverse reactions were aspirin (21), atorvastatin calcium tablets (11), oxiracetam injection (8), TCM decoction (7), metoprolol tartrate (7), insulin (6), deproteinized calf blood extract (6), dexamethasone injection (6), trimetazidine hydrochloride tablets (5), and isosorbide mononitrate (5).

The top 10 injections in combination with SXNI were oxiracetam injection (8), deproteinized calf blood extract (6), insulin (6), dexamethasone injection (6), L-carnitine injection (4), omeprazole injection (4), pantoprazole injection (4), sodium ozagrel (3), bozhi glycopeptide injection (3), and magnesium isoglycyrrhizinate injection (3).

### 3.10 Causal analysis

Age, sex, ethnicity, dosage and twenty-two potential factors contributing to adverse reactions were analysed. There were correlations betweenadverse reactions and sex (*p* = 0.016), storage period (*p* = 0.0015), treatment course (*p* < 0.0001), dripping speed (*p* = 0.0380). Logistic regression was used to analyze the above factors, and the results indicated that the combined significance of the four factors had a significant impact on the occurrence of adverse reactions. At 95% confidence, according to the regression coefficient, women, long dispensing time, short medication course and slow dripping speed rate were considered as risk factors. The causal analysis of ADRs is displayed in Table 7.

**TABLE 7:** Causal analysis of ADRs for patients using SXNI.

Elements	ADR		Correlation with ADRs		
	Yes	No			
Sex [n (%)]	38(70.37)	14484(48.17)	c^2^ test, *p* = 0.016	Regression coefficient, 0.8883	*p* = 0.0030
Famale [n (%)]					
Storage period [min, M±S (%)]	22.85 ± 13.71	17.43 ± 11.96	Permutation test, *p* = 0.0015	Regression coefficient, 0.0243	*p* = 0.0032
Treatment course [d, M±S (%)]	5.00 ± 4.86	9.07 ± 5.56	Permutation test, *p* < 0.0001	Regression coefficient, −0.2189	*p* < 0.0001
Dripping speed [Drips per minute, M±S (%)]	42.28 ± 13.42	46.72 ± 15.69	Permutation test, = 0.0380	Regression coefficient, −0.0246	*p* = 0.0178

## 4 Discussion

### 4.1 The safety result of SXNI

A total of 30,122 patients were included in the prospective hospital-based intensive monitoring study of SXNI, and 54 adverse reactions were identified, with an incidence rate of 0.18%, thereby indicating that the adverse reactions were generally occasional. Most of the adverse reactions of SXNI occurred within 2 h of dosing, mainly manifesting as pruritus, rash and other damage to the skin and its appendages, and the prognosis was good. No serious adverse reactions were observed.

### 4.2 Characteristics of SXNI-related adverse reactions

SXNI-treated patients showing adverse reactions were mostly middle-aged and elderly (over 45 years old), and the proportion of female patients with adverse reactions was higher than that of male patients; 29.63% of patients with adverse reactions had a previous history of food/drug allergy, 27.78% of adverse reactions occurred within half an hour of dosing, and more than half of adverse reactions occurred within 2 h of dosing. Therefore, clinical application of SXNI should be monitored within half an hour of dosing such that adverse events can be handled promptly. Middle-aged and elderly women, especially patients with a history of food/drug allergy, should be under close monitoring to be alert to the occurrence of adverse reactions. Causal analysis showed that women, long dispensing time, short medication course and slow dripping speed rate were considered as risk factors. According to statistic analysis, the shorter the medication course, the more likely ADRs will occur. However, clinically, when patients experience ADRs with SXNI, they generally refuse to use it again, so the occurrence of ADRs may leads to a shortened course of treatment.

### 4.3 Strengths and limitations of the study

This intensive monitoring study of SXNI safety was a prospective observational study with a large sample. The prospective design and quality control process ensured the accuracy and reliability of the study results. This intensive monitoring study covered a period of time from the beginning of drug administration and monitored the incidence of adverse reactions and their clinical manifestations in real-world applications, and was aimed at providing data to support safe applications of SXNI in a clinical setting (Xie et al., 2010; Zheng, 2017). Intensive monitoring is an important supplement to the spontaneous reporting system of adverse drug reactions, and facilitates the detection of unknown or unanticipated adverse drug reactions, thereby allowing for calculation of the incidence of adverse reactions, and compensates for the limitations of insufficient safety assessment in pre-marketing studies (Xie et al., 2019).

However, there is a need to further improve the quality of the study: (1) Given the large number of participating patients and hospitals, the quality control of data collection should be further strengthened; (2) Program training and study implementation should be strengthened to reduce the clinically irrational use of SXNI in healthcare institutions that participate in the intensive monitoring study; and (3) A more in-depth and comprehensive association analysis on the risk factors of adverse reactions is required.

### 4.4 Recommendations for clinical applications

Solvent type and dose are the most easily overlooked yet important factors to consider in a clinical setting. Thus, when applying SXNI in a clinical setting, care should be taken to avoid its application to off-label indications and beyond recommended dosage. Clinicians should be alert to the occurrence of adverse reactions within half an hour of dosing, especially for patients with a history of drug/food allergy. If rash or other discomfort appears, it is required to immediately stop using the drug and provide appropriate treatment. Feedback should be given to the clinician if there is any discomfort within 24 h of dosing.

### 4.5 Recommendations for research and regulation

The requirements for drug risk assessment and management are constantly changing as the methods for clinical safety assessment of drugs are being updated and developed. The new Drug Administration Law of China establishes that the country will implement a pharmacovigilance system to regulate the whole life cycle of drugs. Accordingly, there is an urgent need to upgrade the intensive monitoring research in hospitals by establishing a professional team with the collaboration of multiple participants including government regulators, enterprises, research institutions, and hospitals, to form a long-term, economical, and whole life-cycle monitoring-based safety monitoring model for TCM injections with the aim of achieving routine monitoring of the safety of TCM injections and taking quick actions in response to unexpected safety events. After the completion of this intensive hospital monitoring study of SXNI safety, it is desirable to choose a number of hospitals showing a high degree of cooperation, strict quality control, and high research quality as pilot sites to perform continuous monitoring of the clinical safety of SXNI. The results of an intensive hospital monitoring study should be combined with the results of safety assessment based on literature-reported case data and the results of overall clinical safety assessment based on spontaneous reporting systems. This strategy will provide a basis for pharmaceutical enterprises to revise drug instructions and develop risk control measures. Consequently, this will help incorporate the study results into drug instructions and relevant clinical practice guidelines, which will further guide the rational use of clinical drugs.

## 5 Conclusion

Post-marketing safety surveillance and re-evaluaiton of SXNI included 30,122 patients from 48 hospitals. The incidence of SXNI-related adverse reactions in real-world clinical applications is occasional and in a reasonable range with a good prognosis. Causal analysis showed that women, long dispensing time, and slow dripping speed rate were considered as risk factors. When administering SXNI, physicians should be careful to follow guidelines and package insert.

## Data Availability

The original contributions presented in the study are included in the article/Supplementary Material, further inquiries can be directed to the corresponding author.
